# Appropriate height of dental chairs for effective administration of chest compressions by female dentists

**DOI:** 10.1002/cre2.236

**Published:** 2019-08-16

**Authors:** Kentaro Nogami, Shogo Taniguchi

**Affiliations:** ^1^ Section of Anesthesiology, Department of Diagnostics & General Care Fukuoka Dental College Fukuoka Japan

**Keywords:** cardiopulmonary resuscitation, chest compression, dental chair, female dentist

## Abstract

**Objectives:**

The aim of this study was to determine the appropriate height of a dental chair for the administration of effective chest compressions by female dentists.

**Materials and methods:**

We asked 19 female dentists to perform metronome‐guided chest compressions at a rate of 100 compressions per minute for 2 min on the floor and on a dental chair. We set the height of the dental chair to 76, 73, 70, 67, and 64 cm. We measured the compression depth and proportion of compressions performed at an adequate depth. We then compared the quality of chest compressions between the tall and short (relative to the average body height) groups of participants. We also asked the participants to specify their preferred compression height or condition for chest compression administration.

**Results:**

The participants recorded their maximum chest compression depth (35.0 ± 8.8 mm) at a height of 67 cm. There was no significant difference in chest compression depth between the tall and short groups, irrespective of the compression height. The maximum depth of chest compressions was achieved at a height of 67 cm (from the floor to the compression surface) in both groups, with no significant difference. The participants most frequently identified 67 cm as the most suitable height for the administration of chest compressions.

**Conclusion:**

For female dentists, a height of 67 cm is considered suitable for the administration of chest compressions in the standing position, regardless of physique.

## 1 INTRODUCTION

Proficiency in the administration of cardiopulmonary resuscitation (CPR) is a highly important skill for all health‐care professionals. Recent studies have shown that high‐quality CPR, especially high‐quality chest compressions (CCs; Scapigliati, Ristagno, & Cavaliere, 2013), and early defibrillation are the most important factors for increasing the survival rate. The average citizen is becoming familiar with the concepts of basic life support (BLS), which includes CPR. Accordingly, dentists and para‐dental staff are also becoming more acquainted with the concepts of BLS. This is especially evident from the increasing number of dentists acquiring the American Heart Association (AHA) BLS Healthcare Provider qualification, which is a prerequisite for some dental specializations in Japan. However, our previous study showed that the proficiency of dentists with respect to the BLS Healthcare Provider qualification deteriorates rapidly without retraining (Nogami, Taniguchi, & Ichiyama, 2016). Therefore, we recommend that dentists update their BLS skills periodically. Moreover, it may be effective to revise the BLS course design to include realistic scenarios for dentists (Nogami et al., 2016).

One realistic scenario for dentists is cardiac arrest on the dental chair. If CPR can be performed on a dental chair, it would be unnecessary to lower the victim to the floor before initiating CCs, thus avoiding wastage of time. This would be beneficial for both the rescuer and the victim. A previous study has shown that female dental hygienists can effectively administer CCs on the dental chair or initiate immediate CPR without having to lower the patient to the floor (Yokoyama, Yoshida, & Suwa, 2008). Moreover, the updated European Resuscitation Council guidelines recommend initiating CPR on the dental chair for patients who experience cardiac arrest during dental procedures (Truhlář et al., 2015). The height of the dental chair affects the effectiveness of CCs when CPR is administered on a dental chair. However, there are no studies on the appropriate height for effective administration of CCs. In cases in which dental procedures are performed by female dentists and para‐dental staff, all CPR rescuers may be women. However, there is no study on the appropriate height of dental chairs for the effective administration of CCs by female dentists.

The aim of this study was to determine the appropriate height of a dental chair for the effective administration of CCs by female dentists.

## 2 MATERIALS AND METHODS

Our study participants were 19 female dentists from the Fukuoka Dental College Hospital with BLS training from our postgraduate program but did not have the AHA Healthcare Provider qualification. The mean (± standard deviation) age, height, and weight of the participants were 26 ± 3 years, 157 ± 5 cm, and 50.1 ± 2 kg, respectively. The ethics committee at Fukuoka Dental College determined that ethical approval was not required for this study. All participants provided informed consent for participation in the study.

**Figure 1:**
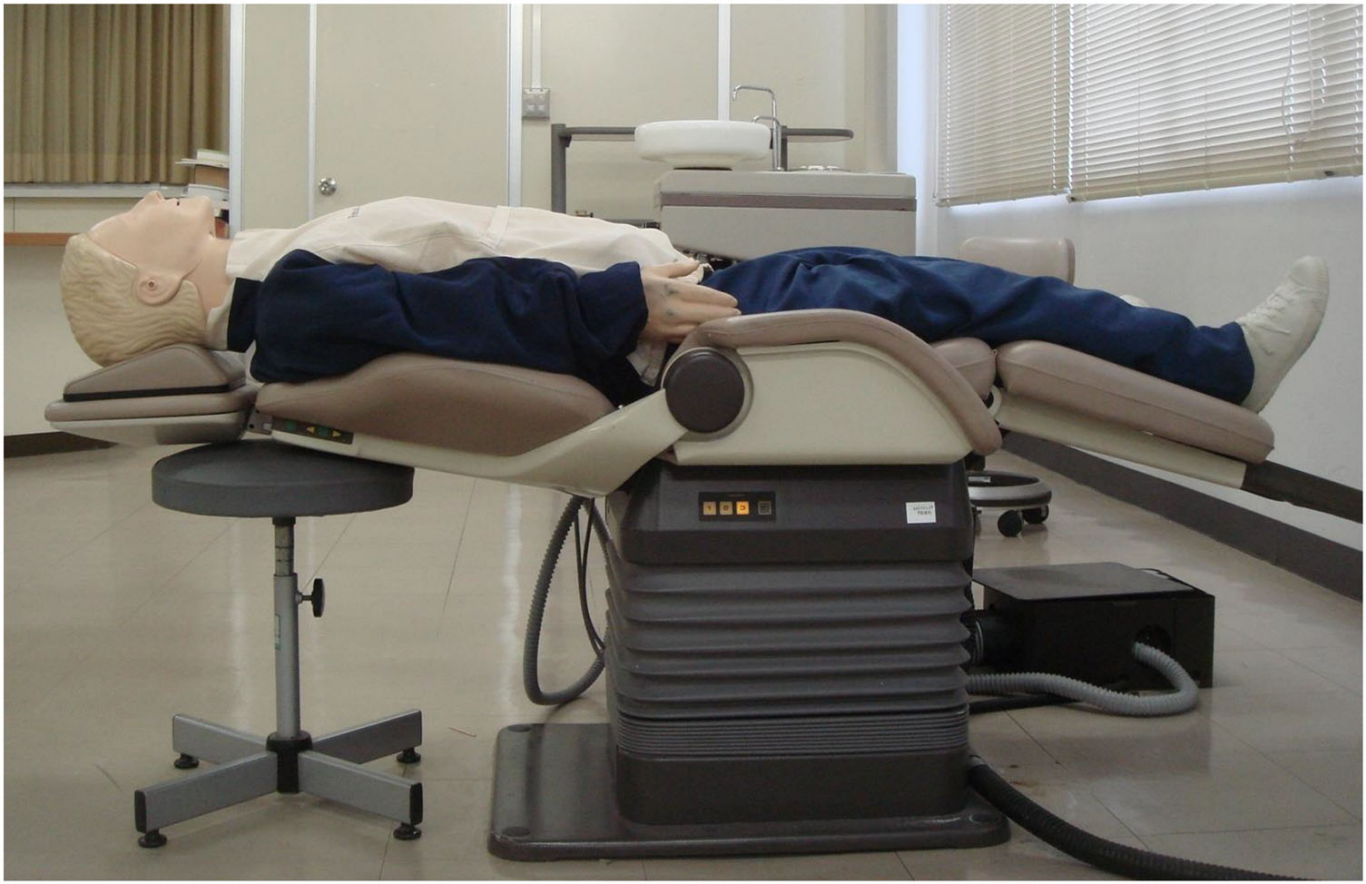
Conditions of chest compression administration. We used a mannequin (Laerdal Resusci Anne) connected to the Laerdal PC Skill Reporting System computer software to evaluate the chest compression quality of the participants. We set the dental chair height (from the floor to the compression surface) to 76, 73, 70, 67, and 64 cm

**Figure 2:**
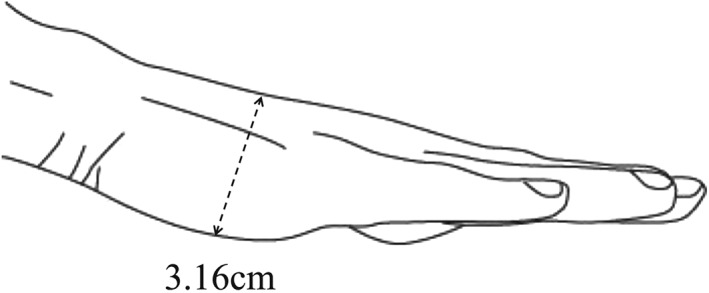
The average hand thickness at the base of metacarpal 5 in Japanese women (cm)

**Figure 3:**
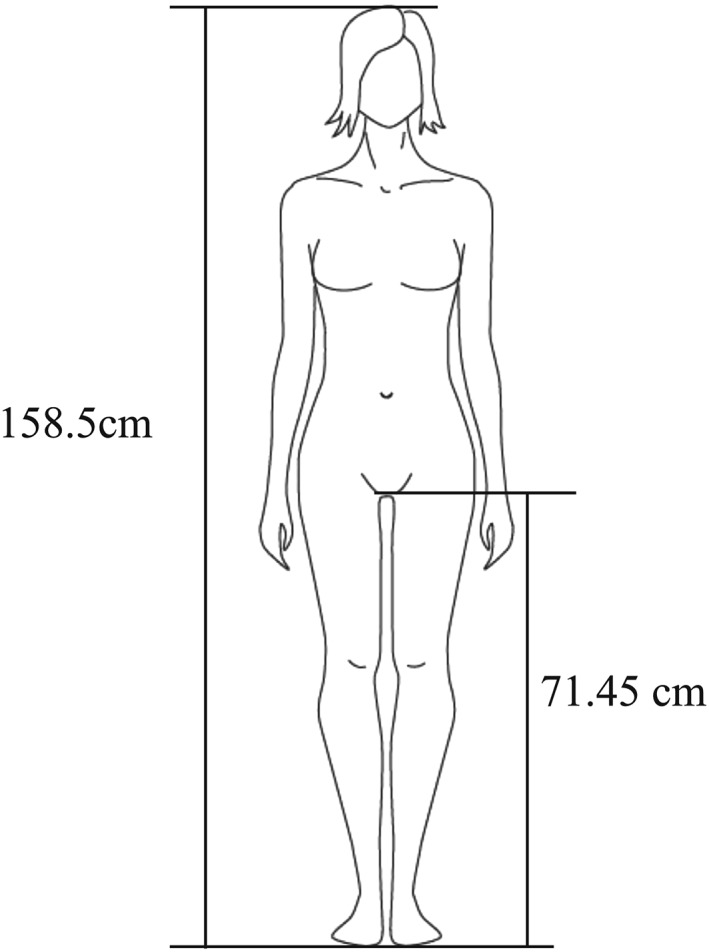
The average inseam length and the average height of Japanese women (cm)

In this study, we used a mannequin (Laerdal Resusci Anne; Laerdal Medical AS, Stavanger, Norway) connected to the Laerdal PC Skill Reporting System computer software to evaluate the CC quality of the participants. We asked the participants to perform CCs for 2 min on the floor and on the dental chair. We set the dental chair height (from the floor to the compression surface of the mannequin) to 76, 73, 70, 67, and 64 cm (Figure 1). The height was set at 3.16 cm on the basis of the average “hand thickness at the base of metacarpal 5″ (i.e., the thickness at the base of the fifth metacarpal when the fingers are straight and the palm is open) in Japanese women (Figure 2; Depth measurements of the hand. D15 hand thickness at metacarpal five base, n.d.). We considered that during stable and effective CC administration, the hands of the rescuer are typically at the level of the beginning of the inseam. The average inseam length or “crotch height” in Japanese women was reported to be 71.45 cm (Figure 3; Crotch height. National Institute of Advanced Industrial Science and Technology (AIST), Digital Human Research Center, n.d.). Therefore, we decided to use a height of 70 cm as the reference point and performed measurements at height increments of 3 cm. Each participant performed CCs at a rate of 100 compressions per minute with a metronome sound guide. We evaluated the following as continuous valuables: CC depth, proportion of CCs of adequate depth, and CC quality of participant groups according to the average body height (tall and short). The adequate CC depth was at least 50 mm. The participants were allowed enough rest between measurements to prevent a reduction in CC quality due to fatigue. The aim of the comparison was to evaluate the correlation between CC quality and the physique of the participants. We divided the participants into two groups: those with greater than average body height (tall group) and those with less than average body height (short group). The average height of Japanese women in their 20s is 158.5 cm (Figure 3). We measured the CC depth and the proportion of adequate CC administrations at the selected heights to determine the preferred height for administration of CCs. Then, we compared the groups to evaluate the CC quality in relation to the physique of the CPR administrator.

In addition, we investigated the preferred height or situation for CC administration through questionnaires completed by all participants.

All statistical analyses were performed using SPSS version 20 (SPSS Inc., Chicago, IL). Students *t* test was used to investigate all measured values. The significance level was set at *p* < .05.

## 3 RESULTS

The participants recorded a mean maximum CC depth of 35.0 ± 8.8 mm at a height of 67 cm from the floor to the compression surface (HFCS; Table 1). However, their CC depth was less than the adequate CC depth. In other words, the proportion of CCs of adequate depth was 0%. The mean CC depth at all heights was 30 mm (Table 1). Because this depth was not effective, we considered a CC depth of 30–50 mm as adequate, as a matter of convenience. The rationale for this is presented in Section 4. We then calculated the proportion of participants able to perform CCs to a depth of 30–50 mm and found that the highest proportion of adequate CCs was reached at an HFCS of 67 cm (Table 2).

**Table 1:** Depth of chest compressions (mm)

	Mean ± *SD*
Floor	33.6 ± 6.8
64 cm	32.8 ± 8.3
67 cm	35.0 ± 8.8
70 cm	33.3 ± 7.6
73 cm	33.2 ± 7.2
76 cm	31.8 ± 8.0

**Table 2:** Proportion of chest compressions of adequate depth (%)

	Proportion ± *SD*
Floor	32.0 ± 32.2
64 cm	18.0 ± 14.0
67 cm	44.0 ± 36.0
70 cm	32.9 ± 38.1
73 cm	27.8 ± 42.0
76 cm	25.1 ± 26.7

There was no significant difference in the depth of CCs performed on the floor or at any HFCS in both groups (Table 3). The maximum CC depth was recorded at an HFCS of 67 cm in both groups, with no significant difference (Table 3).

**Table 3:** Comparison of chest compression depth (mm) between groups according to body height

	Mean in short group ± *SD*	Mean in tall group ± *SD*	*p* value
Floor	31.6 ± 6.8	36.3 ± 6.9	.164
64 cm	31.2 ± 8.4	35.1 ± 8.9	.336
67 cm	33.5 ± 9.2	37.0 ± 8.6	.418
70 cm	32.7 ± 8.0	34.0 ± 7.8	.735
73 cm	32.7 ± 8.2	33.9 ± 6.4	.747
76 cm	31.4 ± 9.6	32.4 ± 6.5	.799

We found that the tall group had a significantly higher proportion of adequate CCs performed on the floor than the short group (*p* = .035); however, there were no significant differences at the other heights at which CCs were performed (Table 4). Both groups showed higher proportions of adequate CCs at an HFCS of 67 cm, but there were no significant differences among the heights (Table 4).

**Table 4:** Comparison of the proportion of chest compressions of adequate depth (%) between groups according to body height

	Proportion in short group ± *SD*	Proportion in tall group ± *SD*	*p* value
Floor	6.2 ± 11.5	52.4 ± 37.0	.035
64 cm	9.2 ± 14.4	24.2 ± 32.1	.238
67 cm	25.8 ± 31.5	57.3 ± 40.2	.083
70 cm	18.9 ± 34.7	43.1 ± 36.3	.164
73 cm	14.4 ± 28.5	37.6 ± 33.4	.132
76 cm	14.8 ± 33.6	32.5 ± 35.4	.261

*Note.* The tall group had a significantly higher proportion of adequate chest compressions performed on the floor than did the short group (*p* = .035).

The preferred heights for CC administration, as indicated by the participants in the questionnaires, were as follows: 67 cm for eight participants, 70 cm for four participants, 73 cm for three participants, 64 cm for two participants, 76 cm for one participant, and on the floor for one participant.

## 4 DISCUSSION

This study showed that the participants preferred an HFCS of 67 cm for a stable posture during CC administration. The most effective CCs were recorded at an HFCS of 67 cm, regardless of body height. This implies that differences in the female physique do not affect the effectiveness of CCs at this HFCS. We assumed that there would be a correlation between CC quality and body size, as the force required to compress the sternum by 5 cm is about 500 N (Chi, Tsou, & Su, 2008; Chi, Tsou, & Su, 2010; Tomlinson, Nysaether, Kramer‐Johansen, Steen, & Dorph, 2007). A previous study on CPR administered by male and female nurses showed that the proportion of effective CCs significantly decreased over time in the light (i.e., low weight) group (Hasegawa, Daikoku, Saito, & Saito, 2014). The CCs in this study were performed on the floor. Similarly, our study showed that participants in the tall group performed significantly higher proportions of effective CCs on the floor than did those in the short group. This means that participants in the short group did not perform as many effective CCs on the floor as did those in the tall group. The correlation between CC quality and body size was considered to be due to the differences in the hip‐to‐knee length, as the CC force generated during CPR is derived from gravity and the hip flexion torque (Trowbridge et al., 2009). The CC force is generated by using gravity to accelerate the upper body downward and using hip extension torque to hold the trunk up at decompression, which resists the inertial force of gravity (Trowbridge et al., 2009). Moreover, the moment of force acting on the lumbar spine represents the load generated by muscles during CCs (Jones & Lee, 2008). An earlier study suggested that at the moment of decompression, the erector spinae muscles generate force that antagonizes compression, which may explain the increased physical fatigue experienced by participants of the light group compared with those of the heavy group (Hasegawa et al., 2014). On the basis of the above‐mentioned reasons, the significant difference that we observed between participants of different body sizes during CCs on the floor may be due to the action of body weight and gravity through the hip joint as the center of moment in a kneeling position. We considered that the body position (standing or kneeling) during CC administration affects the CC quality. An earlier study that investigated body positions during CCs administered by 20 fourth‐year medical students showed no significant differences in the average compression rate or average compression depth between the standing and kneeling positions (Oh, Kim, Kim, Lee, & Lee, 2014). The study suggested that if the height of the bed is adjusted to the level of the rescuer's knees, the CC quality will not differ between the standing and kneeling positions (Oh et al., 2014). However, we postulated that it is more effective to perform CCs in a standing position at a height of 67 cm than in a kneeling position. This is based on our observation that effective CC administration was achieved at an HFCS of 67 cm in both study groups. On account of the above‐mentioned reasons, we considered that it would be easy for female rescuers to harness the force generated by a combination of body weight and gravity for effective administration of CCs at an HFCS of 67 cm.

Our study showed that there was no time‐dependent reduction in CC quality at any of the selected heights. An earlier study that recruited dentistry students showed that the proportion of effective CCs changed with the CC rate between the first and the second sets (Shimizu et al., 2014). In this study, the proportion of CCs of depth > 5 cm performed at a rate of 110 compressions per minute decreased significantly in the second set. However, the study showed that there was no significant difference between the first and second sets at a rate of 100 compressions per minute, which was the same rate used in our study (Shimizu et al., 2014).

Four observational studies published after the publication of the 2010 guidelines suggested that a compression depth in the range of 4.5–5.5 cm in adults yields better outcomes than all other compression depths during manual CPR (Hostler et al., 2011; Stiell et al., 2012; Stiell et al., 2014; Vadeboncoeur et al., 2014). In an analysis of 9,136 patients, compression depths between 40 and 55 mm were associated with the highest survival rates, with a peak at 46 mm (Stiell et al., 2014). However, for convenience, we assumed that a CC depth in the range of 30–50 mm was adequate. Our assumption was based on the results of an earlier study that reported that female rescuers performed CCs to an average depth of 30 mm (Sebbane et al., 2012; Trowbridge et al., 2009). It was presumed that the participants of this earlier study had received sufficient BLS training, as they were emergency department nurses. In contrast, the participants of our study were female dentists who had not received sufficient BLS training nor acquired the AHA Healthcare Provider qualification. Although the CC depth was insufficient according to the guideline recommendations, we considered that this CC depth was adequate for a female rescuer without enough training. Moreover, according to a study on CC quality with real‐time automated feedback, the average CC depths with and without feedback were 38 and 34 mm, respectively (Kramer‐Johansen et al., 2006). On the basis of these results, we considered that female dentists could perform adequate CCs on the dental chair at a height of 67 cm with sufficient training.

## 5 STUDY LIMITATIONS

The data of our study were obtained from Japanese female dentists. Therefore, an HFCS of 67 cm can enable Japanese women of any physique to provide stable CCs in the standing position; however, this height may not be appropriate for female dentists of other races. In other words, the appropriate dental chair height for effective CC administration may be correlated with frame‐like differences in the average height across races. We recognize that this study is limited by its small sample size, which precludes conducting a multivariate analysis of the factors affecting the administration of CCs. In this study, blinding of the participants was impossible; however, the performance of the participants may have been influenced by their personal views on whether CPR should be performed on a dental chair. As none of the participants in our study could adequately perform CCs, female dentists were considered unsuitable for CC administration. However, as mentioned above, they may be able to adequately perform CCs at an HFCS of 67 cm with training that emphasizes upon the use of sufficient force, as female dentists are not limited by gravity or the torque on the trunk. Moreover, the skills emphasized in the new guidelines may be more suitable for persons who seldom perform CPR, such as dentists. Although this study used older guidelines, the same methodology can be applied in future studies that use newer guidelines, the results of which should be compared with those of the present study. In addition, it is necessary to conduct surveys on male dentists in the future.

We did not consider body weight in the physical assessment of the study participants, as there is no correlation between body weight and CC depth, considering that we excluded participants who significantly deviated from the average body weight.

## 6 CONCLUSION

Female dentists are considered to be able to provide stable CCs of adequate depth in the standing position at a height of 67 cm, irrespective of physique. This may be possible if female dentists are sufficiently trained in performing CPR on a dental chair.

## CONFLICT OF INTERESTS

All authors report no conflict of interests related to the manuscript.
